# Halogenated Chrysins Inhibit Dengue and Zika Virus Infectivity

**DOI:** 10.1038/s41598-017-14121-5

**Published:** 2017-10-20

**Authors:** Aphinya Suroengrit, Wanchalerm Yuttithamnon, Pimsiri Srivarangkul, Saran Pankaew, Krongkan Kingkaew, Warinthorn Chavasiri, Siwaporn Boonyasuppayakorn

**Affiliations:** 1 Graduate Program, Faculty of Medicine, Chulalongkorn University, Bangkok, 10330 Thailand; 2 Department of Biology, Faculty of Science, Chulalongkorn University, Bangkok, 10330 Thailand; 3 Department of Chemistry, Faculty of Science, Chulalongkorn University, Bangkok, 10330 Thailand; 4 Chula-Vaccine Research Center, Faculty of Medicine, Chulalongkorn University, Bangkok, 10330 Thailand; 5 Department of Microbiology, Faculty of Medicine, Chulalongkorn University, Bangkok, 10330 Thailand

## Abstract

Dengue virus infection is a global threat for which no specific treatment has not been established. Previous reports suggested chrysin and flavanone derivatives were potential flaviviral inhibitors. Here, we reported two halogenated chrysins, abbreviated FV13 and FV14, were highly potent against DENV1-4 and ZIKV infectivities with the FV13 EC_50_ values of 2.30 ± 1.04, 1.47 ± 0.86, 2.32 ± 1.46, 1.78 ± 0.72 and 1.65 ± 0.86 µM; and FV14 EC_50_ values of 2.30 ± 0.92, 2.19 ± 0.31, 1.02 ± 0.31, 1.29 ± 0.60 and 1.39 ± 0.11 µM, respectively. The CC_50_s to LLC/MK2 of FV13 and FV14 were 44.28 ± 2.90 μM, 42.51 ± 2.53 µM, respectively. Mechanism of drug action studies suggested multiple targets but maximal efficiency was achieved with early post infection treatment. This is the first report showing a high potency of halogenated chrysins for development as a broad-spectrum anti-flaviviral drug.

## Introduction

Dengue virus, a global burden, is a member of the family Flaviviridae and consists of 4 serotypes (DENV1-4). Severe clinical manifestations occur when a previously infected individual becomes infected again with a second, heterotypic virus. The robust but incompetent pathological immune responses^1,2^ can lead to life-threatening conditions like hypovolemic shock, massive bleeding, or multiple organ failure. Until now, the primary treatment of dengue diseases still focuses on supportive therapy and closely monitoring of patients^3^.

Cumulative evidence suggests the level of viral load is associated with progression to severe dengue^4–6^. Therefore, interfering with viral replication is expected to alleviate this critical conditions^7^. Screening and identification of lead compounds *in-vitro* is the first, but crucial, step in the drug discovery pipeline. In recent decades, antiviral drug discovery has exponentially advanced from novel technological developments in both target- and phenotype-based approaches^7^ like molecular docking, *in-vitro* biochemical assays, and cell-based assays. Recently, a live virus assay developed in high-throughput format^8,9^ facilitated the screening of drugs that inhibit the overall viral life cycle. This results of this approach, however, must be confirmed with cytotoxicity counter-screenings.

Small molecules identified as potential flaviviral inhibitors have been originated from various sources such as chemical synthesis, natural extracts, existing compound libraries, or even repurposing of the current drugs. Previous reports suggested that flavonoid derivatives were potential anti-flaviviral active leads^10–13^. Quercetin, a flavonol derivative, was extensively studied as an inhibitor of DENV2 RNA^14^, and DENV2 protease by *in vitro* enzymatic activity and *in silico* molecular docking^12,15,16^. Other flavonoids such as agathisflavone, myricetin^12^, panduratin A^17^ were also reported to inhibit DENV2 protease kinetics and docking. In addition, recent docking studies suggested flavonoids could target DENV envelope (E)^18^ or NS5 RNA-dependent RNA polymerase (RdRP)^19^ proteins. In this study, we explored representatives of flavone, flavanone and chalcone for their activities against dengue and Zika virus, another mosquito-borne flavivirus, in cell culture-based system. Here, we reported for the first time that two chemically modified flavones, halogenated chrysins, were strong candidates for anti-flaviviral replication.

## Results

### Halogenated chrysins, FV13 and FV14, are potential inhibitors of DENV2 infectivity

**Figure 1:**
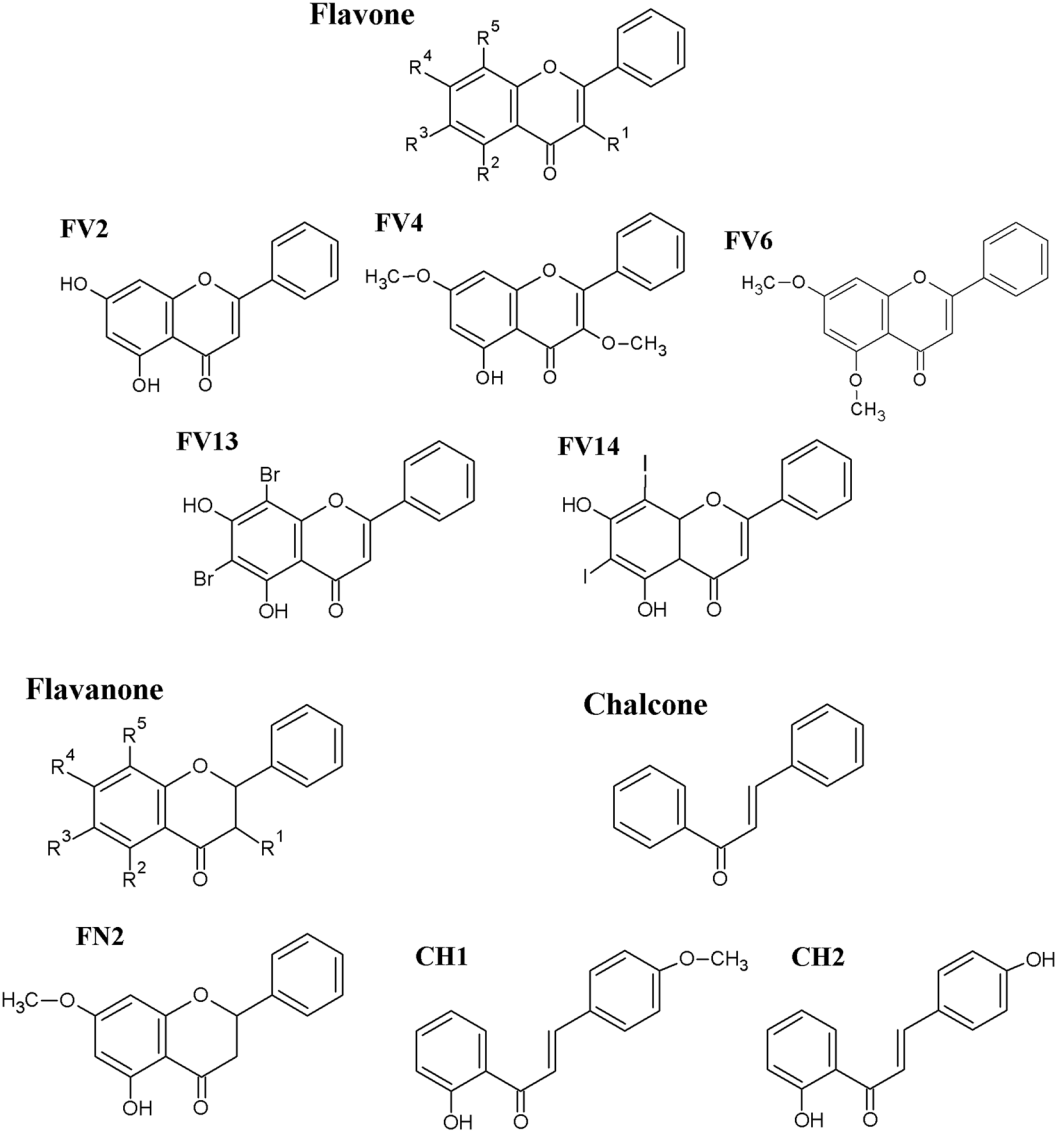
Flavonoid subclasses and structures of tested derivatives.

Previous studies suggested flavone and flavanone derivatives are promising flaviviral inhibitors^10–14,20,21^. In this study, we selected 8 flavonoid derivatives (Fig. 1) and tested their effect on DENV2 NGC in a LLC/MK2 cell-based system. Briefly, the compounds at final concentrations of 10 μM and 25 μM in DMSO were added to DENV2 infected LLC/MK2 cells and the effect on viral particle production was measured by plaque titration of the culture supernatants. Interestingly, two halogenated chrysins, FV13 and FV14, and a chalcone derivative, CH1, strongly inhibited virus production with >99% (Table 1). We also tested cytotoxicity of FV13, FV14, and CH1 to verify the viral inhibition in LLC/MK2 cells (Table 2). The viabilities of FV13 treated cells were 81.00 ± 2.69% and 59.40 ± 2.42%, at 10, and 25 µM respectively, whereas the values for FV14 treated cells were 60.24 ± 3.31% and 60.86 ± 3.57%. We also examined Vero, THP-1, HepG2, and HEK-293 cell viabilities in the presence of selected compounds (Table 2). The results suggested that human-derived cell lines, THP-1, HepG2, and HEK-293 were generally tolerant to the compounds with >85% viability, except for 25 µM CH1 to HepG2 and HEK-293. Vero cells, in contrast, were very sensitive to FV13 and FV14, but not CH1. From this data, we decided to further explore the efficacy of FV13 and FV14 as potential candidates of flaviviral inhibitors.

**Table 1:** Primary screening result in LLC/MK2 cells.

DENV2 inhibition (%, means ± SEM)		
Compounds	**Concentrations**	
	**10 µM**	**25 µM**
**FV2**	43.00 ± 3.00	92.80 ± 3.06
**FV4**	38.00 ± 6.48	61.00 ± 3.00
**FV6**	55.00 ± 3.00	76.00 ± 7.35
**FV13**	99.45 ± 0.48	99.65 ± 0.41
**FV14**	99.48 ± 0.43	99.63 ± 0.03
**FN2**	62.00 ± 2.45	85.00 ± 3.00
**CH1**	99.78 ± 0.08	99.75 ± 0.15
**CH2**	52.00 ± 8.49	76.00 ± 4.24

Means and standard error of the means were calculated from triplicate results.

**Table 2:** Cellular toxicity of selected compounds.

Compounds		Cells viability (%, means ± SEM)				
		LLC/MK2	Vero	THP-1	HepG2	HEK-293
**FV13**	10 μM	81.00 ± 2.69	53.10 ± 4.90	>100	85.83 ± 4.70	98.10 ± 0.72
	25 μM	59.40 ± 2.42	42.57 ± 0.65	86.89 ± 2.10	84.61 ± 2.79	89.43 ± 5.60
**FV14**	10 μM	60.24 ± 3.31	45.44 ± 1.17	86.12 ± 2.76	85.63 ± 3.04	80.47 ± 3.18
	25 μM	60.86 ± 3.57	34.08 ± 2.18	93.92 ± 7.00	87.01 ± 0.52	48.36 ± 5.76
**CH1**	10 μM	61.47 ± 6.43	>100	93.06 ± 4.99	70.97 ± 1.71	98.35 ± 2.95
	25 μM	59.08 ± 7.42	80.44 ± 9.81	84.84 ± 2.38	35.32 ± 1.34	41.26 ± 0.54

Means ± standard error of the means (SEM) of two independent experiments, in which each experiment was performed in triplicate, were reported.

### FV13 and FV14 effectively inhibited all dengue serotypes and Zika virus

**Figure 2:**
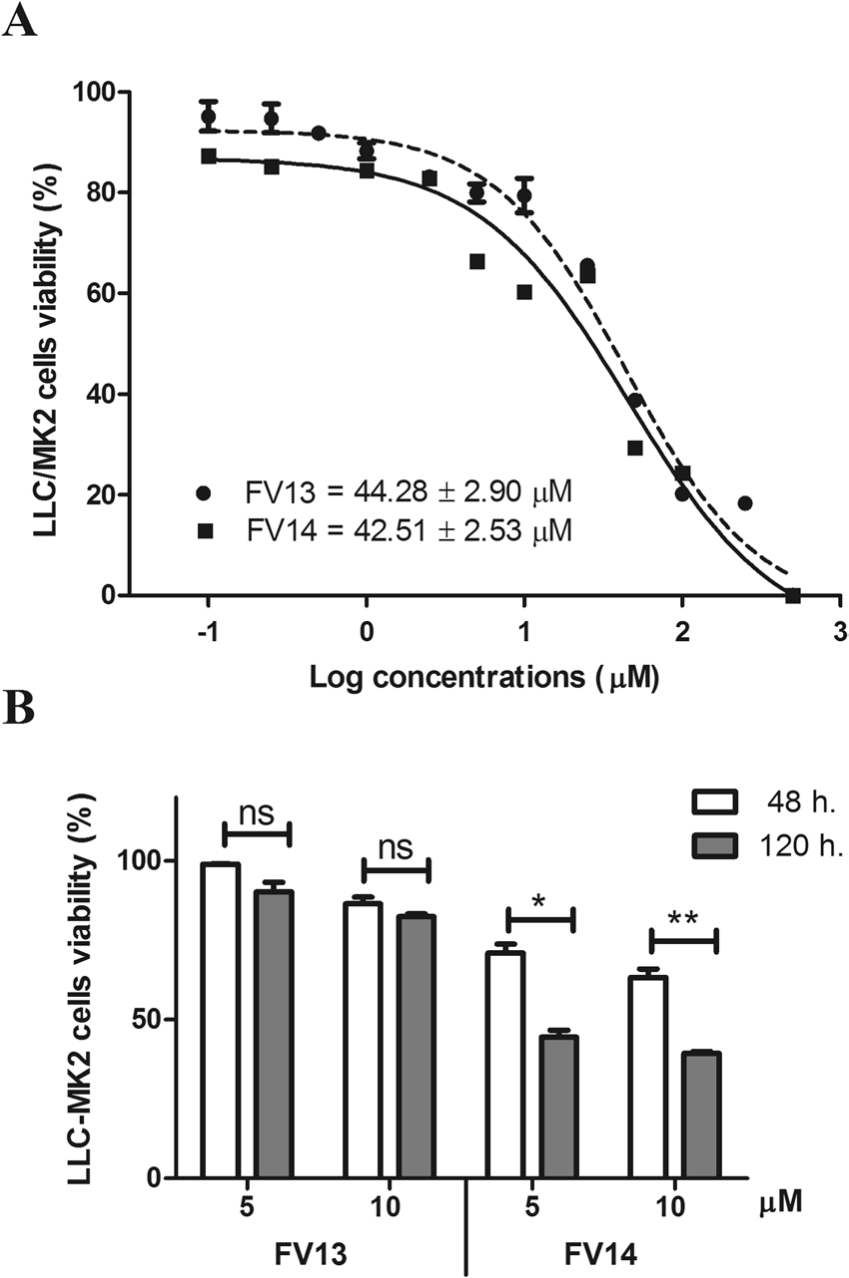
Cytotoxic of two halogenated chrysins. (**A**) Non-linear regression curve and calculated cytotoxicity concentration (CC_50_s) of two halogenated chrysins, FV13 (dash line) and FV14 (bold line), in LLC/MK2 cells at 48 h. (**B**) LLC/MK2 cell viability (%) at 48 h (white bars) and 120 h (gray bars). Values are the means ± standard error of means (SEM) from two independent experiments, in which each experiment was performed in triplicate, were reported. Asterisks indicate statistically significant differences using paired *t*-test as follows; *p-value < 0.05, **p-value < 0.01, ns = no significance.

**Figure 3:**
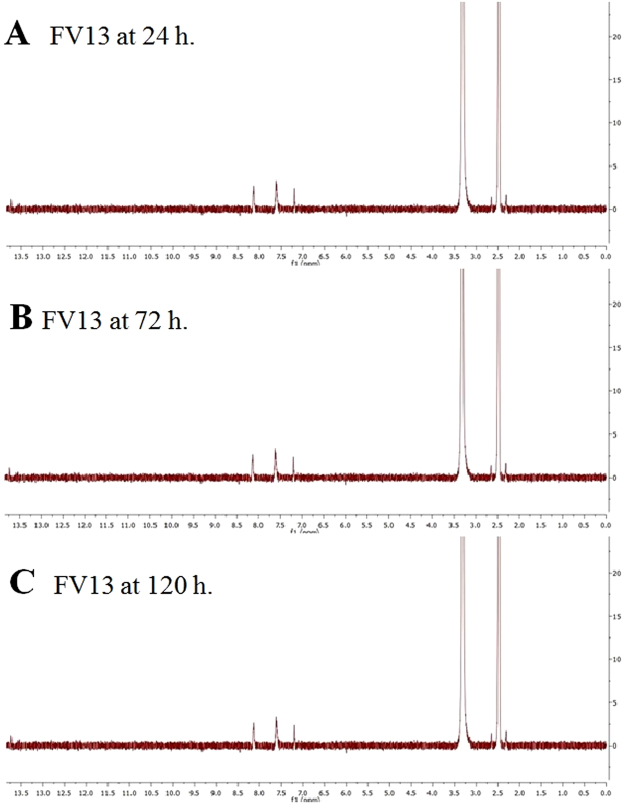
Stability of FV13 following incubation at (**A**) 24, (**B**) 72, and (**C**) 120 h at room temperature.

Next, we examined the efficacy of FV13 and FV14 with DENV1-4 and ZIKV (Table 3). Compound at various concentrations were added to virus infected LLC/MK2 cells and viral titers were accessed by plaque titration of supernatants as previously described. The EC_50_ values of both compounds against DENV1-4 and ZIKV infectivity were ranged between 1–3 µM (Table 3). Note that FV13 and FV14 showed similar efficacy against all tested viruses. Based on these results, we suggested that both compounds could potentially be broad-spectrum flaviviral inhibitors. We further explored cytotoxic concentrations (CC_50_) of FV13 and FV14 to LLC/MK2 cells and both compounds showed similar cytotoxicity after 48 h incubation period at 44.28 ± 2.90 μM and 42.51 ± 2.53 µM, respectively (Fig. 2A). Selectivity indices (CC_50_/EC_50_) (Table 3) indicated that the compounds would not be categorized as drugs with narrow therapeutic index (NTI-drug) and therefore were suitable for further consideration. We also tested the effect of long term incubation on cytotoxicity (Fig. 2B). Whereas FV13 was similarly non-toxic at both 48 and 120 h, FV14 showed significantly greater cytotoxicity at 120 h (*p*-value < 0.01) (Fig. 2B). The limited toxicity of FV13 might reflect rapid degradation of the compound. Therefore, we tested FV13 decomposition in DMSO and incubated at room temperature. FV13 NMR signals at 24, 72, and 120 h (Fig. 3A–C) clearly showed that the compound was stable for at least 120 h at room temperature. Based on the efficacy, toxicity, and stability results, we then chose FV13 to explore its molecular target and mechanism of action.

**Table 3:** Selectivity of halogenated compounds.

Serotypes	Compound			
	FV13		FV14	
	EC50 (µM)^a^	S.I.	EC50 (µM)^a^	S.I.
**DENV1 (16007)**	2.30 ± 1.04	19.42	2.30 ± 0.92	19.39
**DENV2 (NGC)**	1.47 ± 0.86	30.43	2.19 ± 0.31	20.37
**DENV3 (16562)**	2.32 ± 1.46	19.22	1.02 ± 0.31	43.64
**DENV4 (c0036)**	1.78 ± 0.72	25.12	1.29 ± 0.60	34.50
**ZIKV (SV0010/15)** ^**b**^	1.65 ± 0.86	27.02	1.39 ± 0.11	32.14

^a^EC_50_ were determined by plaque assay in 96-well plates.

^b^EC_50_ was determined by plaque assay in 24-well plates.

^c^Selectivity index (CC_50_/EC_50_).

Means ± standard error of the means (SEM) of two independent experiments, in which each experiment was performed in triplicate, were reported.

### FV13 may inhibit multiple targets of the viral life cycle

**Figure 4:**
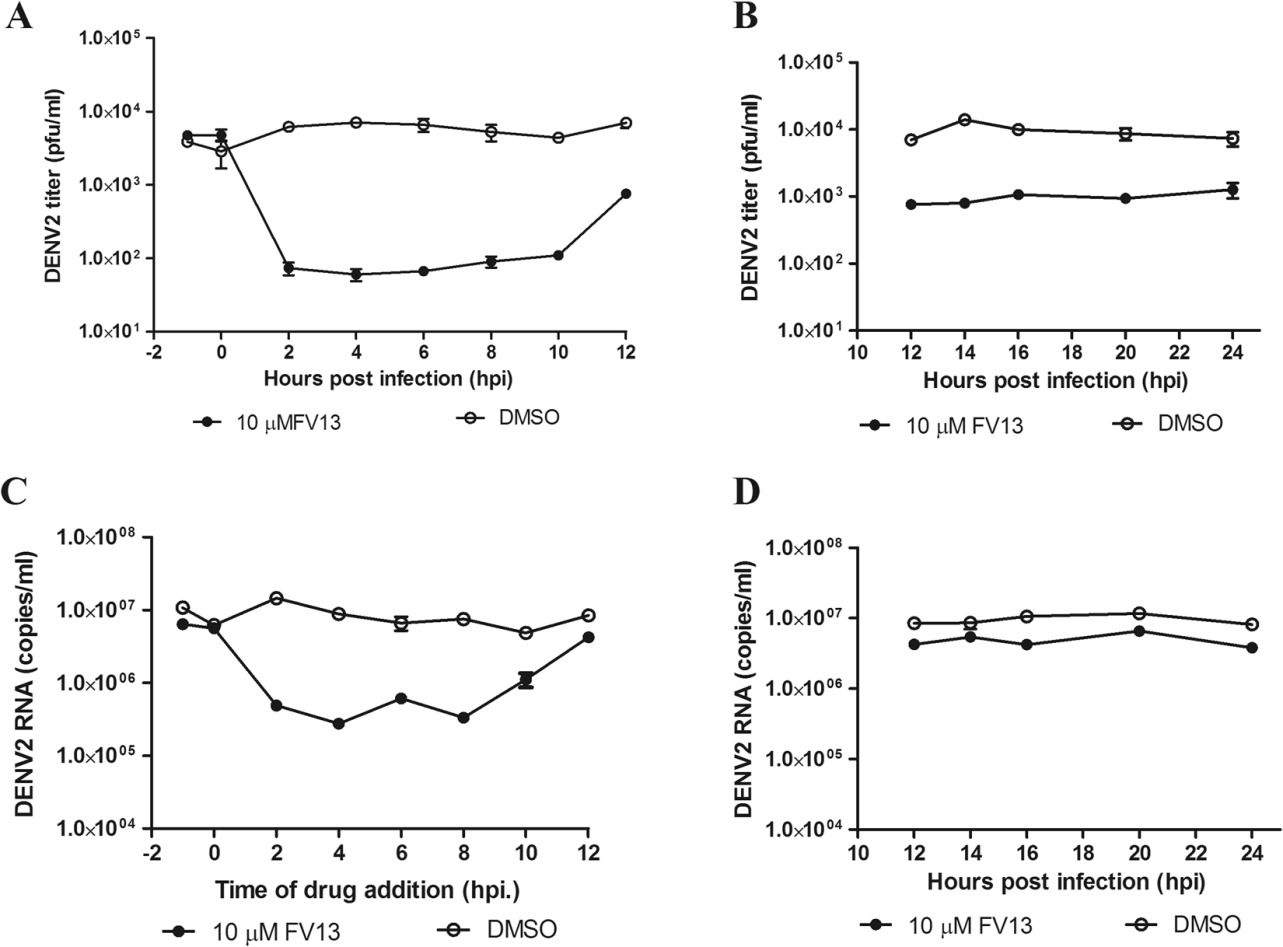
Time-of-drug-addition (TOA) study of FV13 with DENV2 infected LLC/MK2 cells incubated for 120 h. DENV2 plaque titers were from supernatants of cultures in which the drug was added at (**A**) early or (**B**) late times post infection. DENV2 RNA was extracted and quantified from cells of the corresponding cultures at (**C**) early or (**D**) late times.

To access the stage of the viral life cycle affected by FV13, we performed a time-of-addition assay. Briefly, FV13 (10 μM) was added to DENV2 infected LLC/MK2 cells at various time points as described in Methods section. DMSO was added in parallel as a control treatment. Supernatants were collected for plaque titration and cell lysates were collected for RT-qPCR analysis. The titers of FV13-treated samples showed a 2-log decrease from DMSO-treated baseline when the drug was added between 2–10 hours post-infection (hpi). If the drug was added at 12 hpi or later, only 1-log decrease was observed (Fig. 4A,B). The intracellular viral RNA analysis showed a 1-log decrease in FV13-treated samples with drug addition between 2–8 hpi (Fig. 4C). Inhibition gradually declined at 10 hpi and at 12–24 hpi (Fig. 4D), no titer difference was observed between FV13- and DMSO-treated samples suggesting that FV13 did not directly inhibit viral replication. A 1-log difference between plaque and intracellular RNA titers suggests that a post-replication mechanism could be the target of this compound. It also suggests that FV13 may inhibit viral infectivity with at least two mechanisms of action. The first molecular target could interact with FV13 earlier than 2 hpi and its effect was still visible until 8–10 hpi. The other target could locate at late post-replication steps such as assembly, maturation, or release because a 1-log discrepancy between plaque and RT-qPCR titers indicated genome replication was unaffected. Further investigations will reveal insights into the actual target of this compound.

### Maximal efficacy of FV13 is achieved early post-attachment

**Figure 5:**
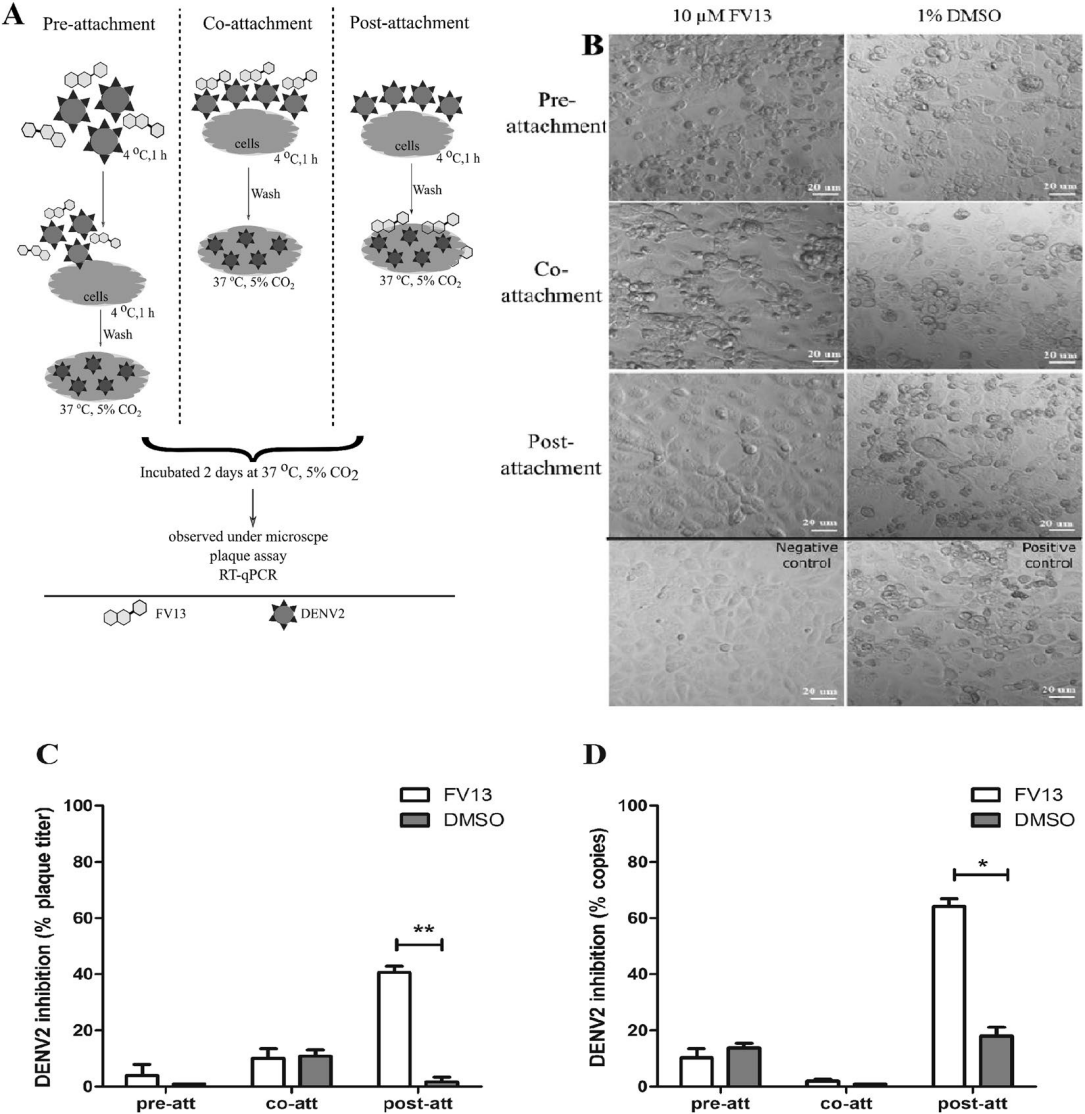
Attachment inhibition study. The assay was performed as illustrated in (**A**). (**B**) LLC/MK2 cytopathic effects were observed at 2 days post-infection under 20x magnification. Scale bars represent 20 µm. (**C**) Plaque titration of supernatants and (**D**) DENV2 intracellular RNAs at pre-, co-, and post-attachment conditions were calculated as percent inhibition of 10 µM FV13 or 1% DMSO treated cells. Means ± standard error of means (SEM) from two-independent experiments, in which each experiment was performed in duplicate, are reported. Asterisks indicated statistical significance using paired *t*-test as follows; *p-value < 0.05, **p-value < 0.01.

We further explored whether the compound would block the viral infectivity by neutralization. We set up the attachment inhibition assay by incubating DENV2 with LLC/MK2 cells at 4 °C for 1 h and FV13 was added before adsorption (pre-attachment), simultaneous with adsorption (co-attachment), and after adsorption (post-attachment) (Fig. 5A). DMSO was used as a mock treatment and added in parallel to FV13 to the experiment. Cells were incubated until cytopathic effects appeared under microscope (Fig. 5B). Supernatants and cells were then analyzed by plaque titration (Fig. 5C) and RT-qPCR (Fig. 5D), respectively. Results indicated that the compound did not interfere with either pre- or co-attachment steps, but rather at post-attachment with plaque titer reductions of 40.59 ± 2.83% (Fig. 5C) and viral RNA reductions of 64.07 ± 3.37% (Fig. 5D). Therefore, the major targets of FV13 inhibition appear to function after attachment, possibly at the fusion or translation steps. Fusion inhibition^22–24^ was subsequently tested with 10 and 25 µM FV13 in DENV2 infected C6/36 cells (M.O.I. of 0.02) but the results showed no difference in the number of fused cells or syncytial phenomenon^25^ (Supplementary 1). Therefore, fusion was unlikely the major target of FV13. We concluded that one of the major targets of FV13 functions early after infection and attachment and fusion are not critically affected. Flavivirus translation initiates within 1 to 5 hpi^26^. Similar to our results, a previously reported translation inhibitor exhibited maximal efficacy early post-infection in a time-of-drug addition profile, and gradually decreasing to no efficacy at 10 hpi^27^.

### Halogenated chrysins inhibited replicon replication

**Figure 6:**
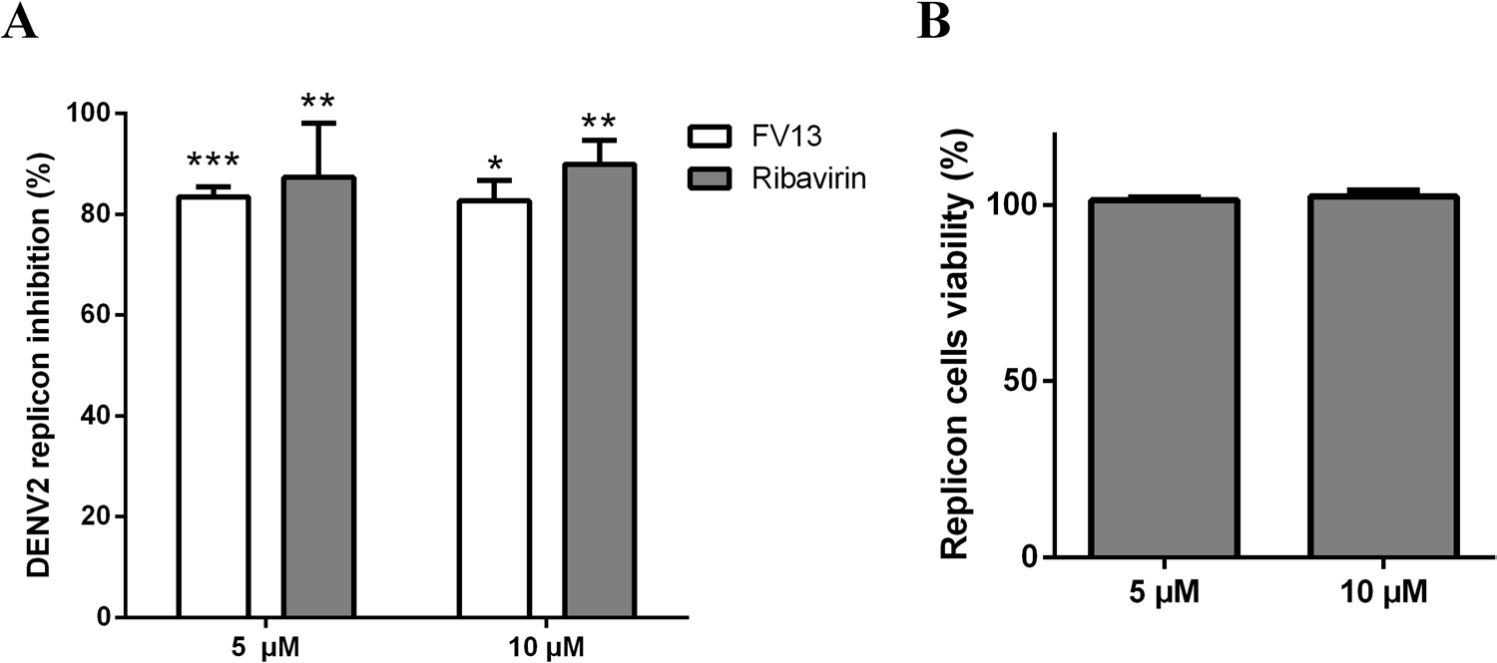
Replicon inhibition study. (**A**) DENV2/BHK-21 replicon cells were treated with FV13 or ribavirin for 72 h. Percent inhibition was calculated from RT-qPCR of extracted RNA. Error bars represented standard error of means (SEM) from two independent experiments with triplicate samples. Asterisks indicated statistical significance in paired *t*-test as follows; *p-value < 0.05, **p-value < 0.01, ***p-value < 0.001 compared to 1% DMSO negative control. (**B**) Percent DENV2/BHK-21 replicon cell viability at 72 h under 5 and 10 µM FV13 treatment. Error bars represented standard error of means (SEM) from three independent experiments, in which each experiment was performed in triplicate.

Next, we studied FV13 inhibition in a DENV2 replicon replication system to investigate whether translation and replication could be implicated as drug targets. BHK-21/DENV2 replicon cells^28^ were treated with 5 and 10 µM FV13 and incubated for 72 h before measuring replication efficiency by quantifying replicon RNA. Ribavirin, a known dengue virus replication inhibitor^29^, was used as a positive control. We found that FV13 efficiently inhibited DENV2 replicon replication by 83.41 ± 2.04%, and 82.71 ± 4.01% when added at 5 µM and 10 µM, respectively (Fig. 6A). The inhibitory effects were similar to those of ribavirin at 5 µM or 10 µM concentrations, 87.37 ± 10.71% and 89.88 ± 4.83%, respectively. Cytotoxicity was also measured and the results showed that most replicon cells (>99%) were viable (Fig. 6B) throughout the experiment. This suggested that FV13 interfered with a sustainable self-replicating viral translation and replication system.

Hundreds of viral and cellular factors are involved in viral translation and replication. We primarily explored DENV protease because previous reports indicated as the target of flavonoid compounds^16,30^. However, our *in vitro* DENV2 NS2B/3 protease assay (Supplementary 2) and molecular docking results (Supplementary 3) indicated that FV13 did not target DENV2 protease. However, several host factors are involved in viral translation step and it is still possible that the compounds target one of these critical factors, subsequently resulting in the inhibition of viral translation.

## Discussion

This is the first report of two halogenated chrysins, FV13 and FV14, showing strong anti-flaviviral efficacies towards the unmodified, naturally derived flavonoids. Active anti-flaviviral (DENV2) compounds accessed by cell-based assay include quercetin (EC_50_ of 118.12 µM)^14^, fisetin (EC_50_ of 192.15 µM)^20^, baicalein (EC_50_ of 23.90 µM)^21^, Naringenin (EC_50_ of 17.97 µM)^10^ and Baicalin (EC_50_ of 30.24 µM)^13^. Obviously, FV13 and FV14 were 20–100 times more potent than previously reported flavonoids. The compounds also showed broad spectrum activities against all dengue serotypes and a Zika virus (Table 3) making them a strong candidate for further drug development.

Moreover, the compounds showed selectivity indices of 20–40, suggesting an applicable therapeutic safety for animal toxicity study. We also examined the cytotoxicity of FV13, FV14 and CH1 to THP-1, HEK-293, and HepG2 cell lines, which are derived from monocytes, renal cells, and hepatocytes, respectively. Results showed that FV13 and FV14 were non-cytotoxic with >80% cell viability (Table 2). Both halogenated chrysins were relatively non-toxic similar to other naturally-derived flavonoids.

Previous pharmacokinetic studies of chrysin in animals and humans showed poor oral bioavailability^31–33^. However, these drugs could be administered by the intravenous route for dengue drug treatment because intravenous fluid replacement is a general practice assigned to patients with impending shock^3^. Moreover, animals can be assigned with broader range of drug since the selectivity indices were 20–40 in cell-based system. Our further investigation will focus on *in-vivo* toxicity and efficacy studies by examining toxic metabolites, half-life of the drug excretion, and monitoring liver and kidney functions.

Undoubtedly, the halogens at the R3 and R5 positions (Fig. 1) convey strong biological activities to FV13 and FV14 against flaviviral replication. Moreover, we noticed that FV2, a naturally purified flavone and a precursor of FV13 and FV14, actively inhibited DENV2 at 10 and 25 µM with 43.00% and 92.80%, respectively. Two hydroxyl groups at R2 and R4 positions were noted as common characteristics of the FV2, FV13, FV14, as well as quercetin, fisetin, baicalein, luteolin, apigenin, etc. Therefore, the hydroxyl groups at R2 and R4 and the high EN groups at R3 and R5 positions could be responsible for crucial biological activity towards flaviviral infectivity.

In search of molecular targets and mechanisms of drug action, a series of experiments directed us to at least two targets located at early and late steps of infection. Although the drug targets are still elusive, evidences suggested viral translation or replication^34–36^. Possible targets could also locate at post-replication steps given a 1-log difference between plaque titers and intracellular viral copies (Fig. 4A–D). Multiple viral and host proteins involved in translation or replication are possible compound targets. Further investigation (e.g. chemical affinity-tag purification coupled with identification by liquid chromatography and mass spectrophotometry; western blot analysis for viral protein expression; and escape mutant study by whole genome sequencing) are methods of choice to identify the drug target.

In conclusion, two halogenated chrysins were demonstrated for the first time as potential inhibitors of dengue and Zika infectivity with high efficacy and low cytotoxicity.

## Materials and Methods

### Compound synthesis, purification and stability

Eight flavonoid derivatives consisting of five flavones (FV), one flavanone (FN), and two chalcones (CH) were selected as representatives for primary screening. FV2 (chrysin) was purchased from Sigma Aldrich, St. Louis, USA. FV4 was extracted and purified from *Kaempferia parviflora*. FV6 was chemically modified from FV2 by methylation. FN2 was extracted and purified from *Boesenbergia rotunda*. CH1 and CH2 were purchased from Sigma Aldrich, St. Louis, USA. The purity and identity of each compound was verified by ^1^H-NMR Tensor 37 infrared spectrometer (Bruker, Massachusetts, USA).

The compound FV13 (6,8-dibromo-5,7-dihydroxyflavone) was synthesized and purified according to the established protocol^37^. Briefly, chrysin (5,7-dihydroxyflavone) (Sigma Aldrich, St. Louis, USA) and NaBr (Sigma Aldrich, St. Louis, USA) were simultaneously dissolved in acetones-water (5:1) (RCI labscan, Samutsakorn, Thailand). The suspension was incubated at room temperature overnight with a continuous stirring. FV13 was identified by thin-layer chromatography (Merck, California, USA) with a hexane-ethylacetate (3:2) solvent system and subsequently crystalized using absolute methanol (RCI labscan, Samutsakorn, Thailand). The pale green solid (68%) was collected and the compound identity and purity were verified by 1H-NMR (Tensor 37 infrared spectrometer) at (400 MHz, DMSO-d6) δ 13.65 (s, 1 H), 8.05 (d, J = 7.4 Hz, 2 H), 7.56 (d, J = 7.5 Hz, 3 H), 7.07 (s, 1 H).

The compound FV14 (6,8-diiodo-5,7-dihydroxyflavone) was synthesized and purified according to the established protocol^38^. Briefly, chrysin dissolved in glacial acetic acid and NaI (May&Baker, London, UK) dissolved in CH_2_Cl_2_ were mixed together. The pale yellow solid (77%) was recovered and the compound identity and purity were verified by 1H-NMR (Tensor 37 infrared spectrometer) at (400 MHz, DMSO-d6) δ 8.18 (d, J = 7.4 Hz, 2 H), 7.60 (d, J = 8.7 Hz, 3 H), 7.17 (s, 1 H).

The FV13 (6,8-dibromo-5,7-dihydroxyflavone) was tested for the stability by dissolving in DMSO at a concentration of 4 mM FV13 solution. The solution was incubated at room temperature and sampled out at 24, 72, and 120 hours for ^1^H-NMR analysis.

All compounds were stored as solid powder at room temperature. The stock solutions (50 mM) were prepared in dimethyl sulfoxide (DMSO) (Merck, California, USA) and stored as aliquots at −20 °C until use.

### Cells and Viruses

LLC/MK2 (ATCC^®^ CCL-7) and C6/36 (ATCC^®^ CRL-1660) cell lines were maintained in minimal essential medium (MEM) (Gibco^®^, Langley, USA) supplemented with 10% fetal bovine serum (Gibco^®^, Langley, USA), 100 I.U./ml penicillin (Bio Basic Canada, Ontario, Canada), and 100 μg/ml streptomycin (Bio Basic Canada, Ontario, Canada), and 10 mM HEPES (4-(2-hydroxyethyl)-1-piperazineethanesulfonic acid) (Sigma Aldrich, St. Louis, USA) at 37 °C under 5% CO_2_ and 28 °C, respectively. HepG2 (ATCC^®^ HB-8065) and HEK-293 (ATCC^®^ CRL-1573) cell lines were maintained in Dulbecco’s Modified Eagle Medium (DMEM) (Gibco^®^, Langley, USA) supplemented as previously described. Vero (ATCC^®^ CCL-81) cell line was maintained in Medium 199 (Gibco^®^, Langley, USA), and THP-1 (ATCC^®^ TIB-202) cell line was maintained in RPMI-1640 (Gibco^®^, Langley, USA) supplemented as previously described. Reference strains of DENV1 (16007), DENV2 (New Guinea C strain), DENV3 (16562), and DENV4 (c0036) were propagated in Vero cell line with Medium-199 supplemented with 1% fetal bovine serum, 100 I.U./ml penicillin, 100 μg/ml streptomycin, and 10 mM HEPES at 37 °C under 5% CO_2_. Zika virus (SV0010/15) was propagated in C6/36 cells^39^ with MEM supplemented as previously described and maintained at 28 °C.

### Antiviral efficacy of the selected flavonoids

LLC/MK2 cells were seeded at 5 × 10^4^ cells into each well of 24-well plate and incubated overnight at 37 °C under 5% CO_2_. Cells were infected with DENV2 at the multiplicity of infection (M.O.I.) of 0.1, unless indicated otherwise, for 1 h with gentle rocking every 15 min. Cells were washed with phosphate buffer saline (PBS) and incubated with 1 ml of MEM medium supplemented with 1% FBS as previously described. The compounds were prepared in dimethylsulfoxide (DMSO) at the indicated concentrations before addition to viral infected cells. Supernatants were collected after 5 day incubation and analyzed by plaque titration^9^. In primary screening, DMSO-treated cells served as the untreated control. Compounds that inhibited virus production by ≥90% were selected for further characterization.

Compounds were further analyzed for their effective concentration (EC_50_) against DENV1-4 and ZIKV. Compounds were prepared in DMSO at 8–12 different concentrations and added to virus infected cells as previously described. Supernatants were collected and the EC_50_ values were calculated from nonlinear regression analysis. Results were reported as means and standard error of the means (SEM) of EC_50_ values from at least two independent experiments in which each drug concentration was tested in triplicate.

### Cytotoxicity study

The cytotoxicity of active compounds was tested on LLC-MK2, Vero, THP-1, HEK-293, HepG2 and C6/36 cell lines. Each cell line was seeded at 1 × 10^4^ cells per well in 96-well plates and incubated overnight. The compounds were added at the indicated concentrations and the cells were incubated for 48 h, unless indicated otherwise. Cell viability was analyzed using CellTiter 96^®^ AQueous One Solution Cell Proliferation Assay kit (Promega, Wisconsin-Madison, USA) according to manufacturer’s protocol. The plate was read at the *A*_450_ in a VICTORTM × 3 microplate reader (PerkinElmer, Massachusetts, USA). Percent cell viability in compound-treated wells was calculated using reference DMSO-treated cells as a 100% cell viability control. The CC_50_ values of LLC/MK2 cells were calculated using nonlinear regression analysis. Results were reported as means and standard error of the means (SEM) of CC_50_ values of three independent experiments in which each drug concentration was tested in triplicate.

### Reverse transcription and quantitative polymerase chain reaction

After supernatant collection, the remaining cells in experimental plates were extracted for viral RNAs using TRIzol reagent (Invitrogen, California, USA) according to the manufacturer’s protocol. The samples were loaded into the Direct-zol™ RNA MiniPrep (Zymo research, California, USA) and quantified by Nanodrop spectrophotometry (Eppendorf, New York, USA). The RT-qPCR was performed with a Step-OnePlus Real-Time PCR System (Applied Biosystems, California, USA.) with 1 × Power SYBRGreen PCR Master Mix, 400 nM each of capsid primers^40^ or 250 nM of NS1 primers^41^ and 0.1 µg of total RNA. The reactions were then cycled at 48 °C 30 min and 95 °C 10 min, followed by 45 cycles of 95 °C for 20 s (denaturation), 55 °C for 30 s (annealing), 72 °C for 30 s (extension). Each sample was analyzed in triplicated and results were confirmed by two independent experiments.

### Time-of-drug addition study (TOA)

LLC/MK2 cells were seeded in 24-well plate and incubated as previously described. Cells were then infected with DENV2 (M.O.I. of 0.1). FV13 (10 µM) was added at various time points, early time points (−1, 0, 2, 4, 6, 8, 10, and 12 hpi) and late time points (12, 14, 16, 20, and 24 hpi) and the plates were incubated for 5 days. Supernatants were collected to determine the viral titer by plaque titration assay and the cells were collected to determine viral RNA content by RT-qPCR. Results were confirmed by three independent experiments in which each time point was tested in duplicate.

### Attachment inhibition study

The attachment assay was adapted from Lani *et al*., 2016 and Jin *et al*., 2015 ^42,43^. Briefly, LLC/MK2 cells were seeded in 24-well plates and incubated as previously described. Cells were then adsorbed by DENV2 (M.O.I. of 1) diluted in maintenance medium at 4 °C for 1 h with continuously gentle rocking. FV13 at 10 µM was added to DENV2 virus preparation for 1 h before adsorption (pre-attachment), during adsorption (co-attachment), and after adsorption plus three washings with cold PBS to remove external viruses (post-attachment). Cells were incubated in maintenance medium at 37 °C, under 5% CO_2_ for 2 days before supernatants and pellets were collected. DMSO-treated samples were used as a no-inhibition control. Pictures were taken using an Eclipse TS100 Inverted Routine Microscope (Nikon, New York, USA). Results were confirmed by three independent experiments.

### Replicon inhibition assay

BHK-21 cells expressing a DENV2 replicon (BHK-21/DENV2) were maintained in minimal essential medium (MEM) supplemented with 10% fetal bovine serum (FBS), and 0.3 mg/ml G418 (Bio Basic Canada, Ontario, Canada). The protocol was adapted from Boonyasuppayakorn *et al*., 2014 ^28^. Briefly, cells (5 × 10^4^/well) were seeded into 24-well plates and were incubated for 1 day at 37 °C in a humidified CO_2_ chamber followed by addition of the compounds in 1% DMSO at final concentrations of 5 µM and 10 µM. DMSO (1%) alone was used as the no-inhibitor control (0% inhibition) and the reference compound was ribavirin (TargetMol, Massachusetts, USA) at a final concentration of 5 µM or 10 µM. Cells were incubated at 37 °C for 72 h and lysed to quantify DENV2 replicons by RT-qPCR. Data were reported as percent inhibition compared with ribavirin, as a reference compound and DMSO, 0% inhibition. Results were confirmed by three independent experiments.

### Data Availability

All data generated or analyzed during this study are included in this published article and supplementary file.

### The level of bio-containment

We performed all pathogen-related experiments in bio-containment level 2. BSL-2 standard operating procedure was performed to fit the requirement of ISO15189 and ISO15190.

## Electronic supplementary material

Supplementary Information
