# Supplementary material for: Halogenated Chrysins Inhibit Dengue and Zika Virus Infectivity
Source: Sci Rep. 2017 Oct 20;7:13696. doi: 10.1038/s41598-017-14121-5 (PMC5651866; doi:10.1038/s41598-017-14121-5)
Supplement: Supplementary file 1 — Supplementary Information [file 41598_2017_14121_MOESM1_ESM.pdf]

Supplementary: Halogenated Chrysins Inhibit Dengue and Zika Virus Infectivity

Aphinya Suroengrit[1], Wanchalerm Yuttithamnon[2], Pimsiri Srivarangkul[2], Saran Pankaew[2], Krongkan Kingkaew[3], Warinthorn Chavasiri[3], and Siwaporn Boonyasuppayakorn[4,5*]

[1]Graduate Program, Faculty of Medicine, Chulalongkorn University, Bangkok, 10330, Thailand

[2]Department of Biology, Faculty of Science, Chulalongkorn University, Bangkok, 10330, Thailand

[3]Department of Chemistry, Faculty of Science, Chulalongkorn University, Bangkok, 10330, Thailand

[4]Chula-Vaccine Research Center, Faculty of Medicine, Chulalongkorn University, Bangkok, 10330, Thailand

[5]Department of Microbiology, Faculty of Medicine, Chulalongkorn University, Bangkok, 10330, Thailand

*Corresponding Author

Supplementary 1

Fusion inhibition study

The assay protocol was adapted from Ichiyama *et al*., 2013 and Poh *et al*., 2009[1,2]. C6/36 cells were seeded at $2x10^5$ cells per well in 24-well plate and incubated overnight. Cells were infected with DENV2 (M.O.I. of 0.02) at 28 ^oC for 1 h with gentle rocking every 15 min. FV13 at 10 µM was introduced to the cells during and after infection. DMSO was added to the infected cells as a no-inhibition control and 4G2 was incubated with DENV2 at 37 ^oC 1 h prior infection, as a positive- inhibition control to observe fusion. On the second day of incubation, maintenance media was adjusted by 0.5 M N-morpholino ethanesulfonic acid (MES) (Sigma Aldrich, St. Louis, USA) to pH~5.5. The cells were closely monitored under microscope. Pictures were taken using Eclipse TS100 Inverted Routine Microscope (Nikon, New York, USA). Results were confirmed by three independent experiments.

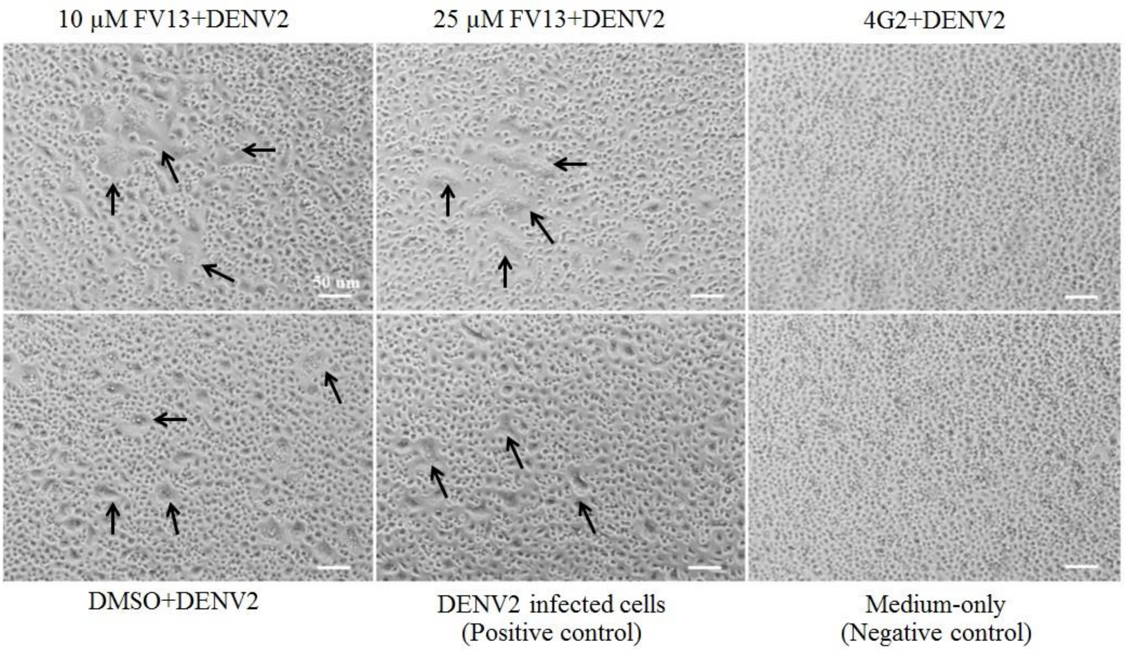

Morphology of DENV-infected C6/36 cells that were treated with 10, 25 µM FV13, 4G2, and 1%DMSO before induced cell fusion with MES. The fused cells (arrow) were observed after 48 h. Positive control was DENV2-infected C6/36 cells and negative control was C6/36 cells in maintenance medium. Scale bars was represented to 50 micrometers.

Supplementary 2

<u>*In vitro* Protease assay</u>

Assays were performed in triplicate in a 96-well half area black plate (Greiner Bio-One, Monroe, NC, USA). The reaction mixture (100 µl) contained 200 mM Tris-HCl, pH 9.5, 30% glycerol, 0.1% CHAPS, 1% DMSO, 50 nM DENV2 NS2BH-(QR)-NS3pro enzyme[3], 10 µM fluorogenic tetrapeptide substrate, Bz-Nle-Lys-Arg-Arg-AMC, and designated concentrations of compounds in DMSO (concentration of 1%). The compound-enzyme mixture was pre-incubated for 15 min at room temperature before addition of the substrate. The reaction was continued at 37 °C for 30 min. The release of AMC from the substrate was recorded every 1.5 min at 380 nm excitation and 460 nm emission in a SpectraMax Gemini EM spectrofluorometer (Molecular Devices, Sunnyvale, CA). DMSO alone (concentration of 1%) was used as the no-inhibitor control (100% protease activity) and the bovine pancreatic trypsin inhibitor (BPTI, also known as aprotinin), which has a Ki of 26 nM against the DENV2 protease, was used at 5 µM in DMSO concentration of 1% as a positive control (0% protease activity). Data were plotted and reported as percent inhibition of each compound to protease activity.

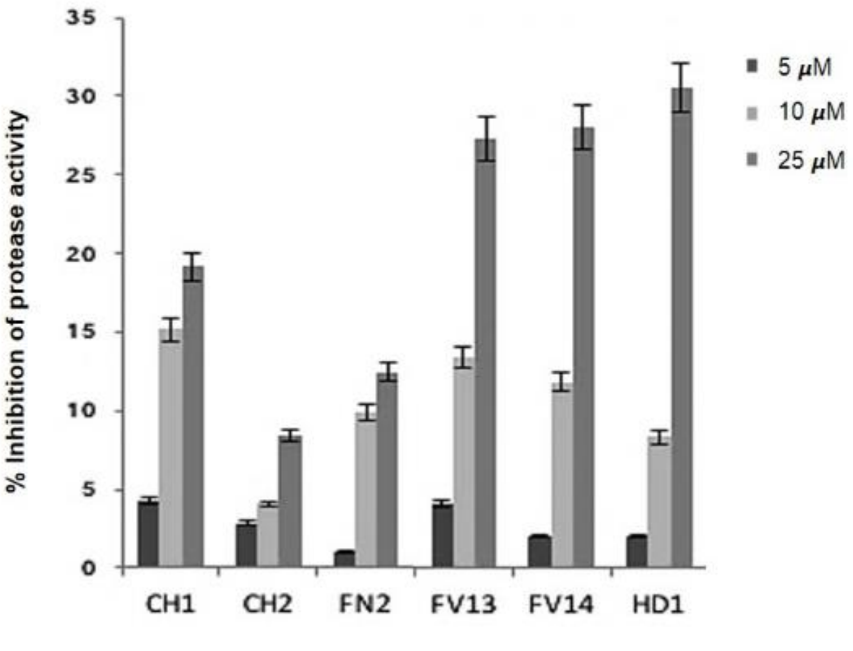

The *in vitro* enzymatic protease assay. Compounds were prepared three concentrations and mixed with protein, added substrate (Bz)-Nle-Arg-arg-AMC and measured at 460 nm with an excitation at 380 nm. DMSO control represented as 100% activity.

Supplementary 3

<u>NS2B/NS3 protease docking study</u>

Briefly, the structure of compound structures were drawn by using HyperChemTM (Hypercube, Inc.) and optimized with molecular mechanics method for geometry optimization. For the NS2B-NS3 protease structure was solved by crystallized structure of crystal structure of DENV3 NS2B-NS3 in complex with aldehyde inhibitor Bz-nKRRR-H (pdb: 3U1I) was used as representative of DENV protease. The structure of protein was visualized by Visual Molecular Dynamics software (VMD) and used Autodock4 (The Scripps Research Institute, US.) to perform molecular docking. The result was obtained in sets of binding and estimated free energy.

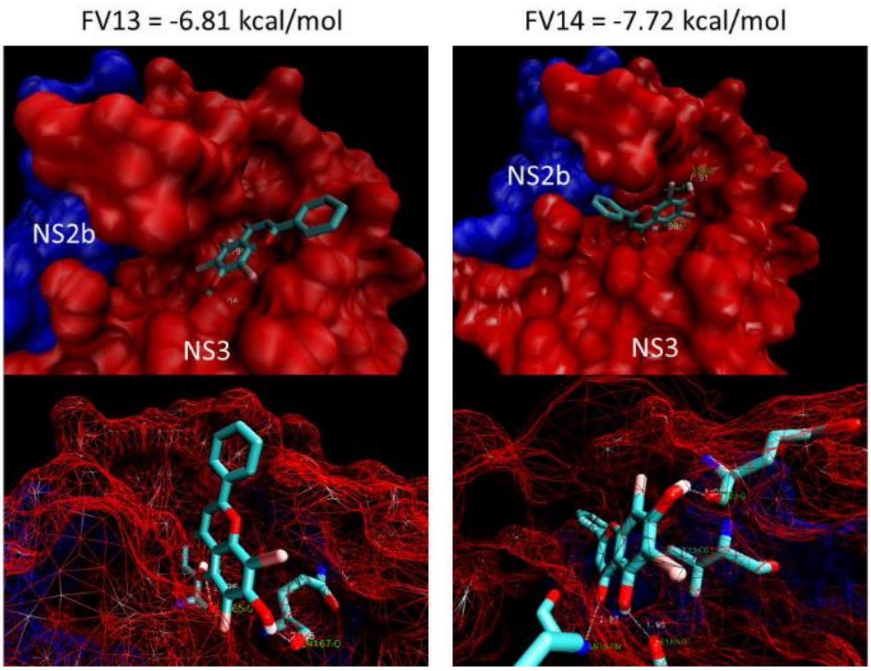

Schematic of the DENV2 NS2B/3 protease with FV13 and FV14 docking at the allosteric site of viral protease.

Reference

1 Ichiyama, K. *et al.* Sulfated polysaccharide, curdlan sulfate, efficiently prevents entry/fusion and restricts antibody-dependent enhancement of dengue virus infection in vitro: a possible candidate for clinical application. *PLoS Negl Trop Dis* 7, e2188, doi:10.1371/journal.pntd.0002188 (2013).

2 Poh, M. K. *et al.* A small molecule fusion inhibitor of dengue virus. *Antiviral Res* 84, 260-266, doi:10.1016/j.antiviral.2009.09.011 (2009).

3 Yon, C. *et al.* Modulation of the nucleoside triphosphatase/RNA helicase and 5'-RNA triphosphatase activities of Dengue virus type 2 nonstructural protein 3 (NS3) by interaction with NS5, the RNA-dependent RNA polymerase. *The Journal of biological chemistry* 280, 27412-27419, doi:10.1074/jbc.M501393200 (2005).
